# A scoping study to explore the potential for developing a feasibility and acceptability pilot of the interRAI Self-reported Assessment of Mental Health (SAMH) with adults experiencing homelessness

**DOI:** 10.1192/j.eurpsy.2026.11303

**Published:** 2026-07-31

**Authors:** C. L. Rose, N. Lockwood, H. Van Marwijk, A. Aslan, D. Haywood

**Affiliations:** Brighton and Sussex Medical School, Brighton, United Kingdom

## Abstract

**Introduction:**

People experiencing homelessness (PEH) are disproportionately affected by poor physical and mental health, frequently having unmet needs. This target group often have complex, multifactorial cases, and experience several barriers to accessing and accepting care. Flexible and low-pressure self-assessment methods might help with the identification and monitoring of mental health conditions for PEH. Community workers have insight into the needs and communication styles of PEH.

**Objectives:**

This study aims to gather feedback on the interRAI Self-reported Assessment of Mental Health (SAMH) from community workers with experience of working with PEH, to support the future development of feasibility and acceptability studies on the integration of the interRAI SAMH into existing care provision for PEH.

**Methods:**

This study takes a qualitative approach, using semi-structured interviews to explore the perceptions of useability and acceptability of the interRAI SAMH for use with the homeless population, reported by four adult community workers, who have experience supporting the mental health and care needs of PEH. The interviews were coded and analysed in line with accepted principles of thematic analysis.

**Results:**

Preliminary results indicate some structural and stylistic barriers to completion of the interRAI SAMH, such as the length, language and formality as well as factors particular to PEH like accessibility. Additional issues related to service eligibility and lack of mental health resources for use after completing the interRAI SAMH. In terms of the content, findings suggest the interRAI SAMH addresses a sufficiently comprehensive range of mental health and social care needs of relevance to PEH.

**Conclusions:**

With adjustments, the interRAI SAMH presents a potentially useful resource for the holistic assessment of the mental health and care needs of PEH. Revising the phrasing of some questions to enhance readability by simplifying language used is one aspect of the questionnaire that was identified to increase the accessibility of the questionnaire for PEH. In terms of the development of a follow-on study on designed to directly address the feasibility and acceptability of the interRAI SAMH for PEH themselves, findings suggest that this should be done alongside the support of trusted and experienced community workers and/or with PEH with greater levels of independence and stability.

**Disclosure of Interest:**

None Declared

